# Mr. E. Chatelier

**Published:** 1910-06-25

**Authors:** 


Mr. E. Chatelier, the Senior Medical Superintendent of the City Hospital, Birmingham, has died, at the age of forty-two, somewhat suddenly. His education included three years in the medical college at Madras, and he completed his course at Edinburgh, taking his M.B. and C.M. degrees in 1893. Later he was appointed assistant medical officer at the Lodge Road City Hospital, Birmingham, in 1896, and in the same year medical superintendent of the Little Bromwich Hospital. When the Lodge Road Institution reopened he had charge of the diphtheria and typhoid fever wards. This experience was invaluable to him as a medical officer.

